# Pandemic-related changes in seasonal vitamin D disparities among preschool children: a community-based cross-sectional study

**DOI:** 10.3389/fnut.2026.1893922

**Published:** 2026-08-17

**Authors:** Yue Bi, Qiao Wang, Jiaxing Yi, Jishan Zheng, Hehe Chen, Wenyuan Liu

**Affiliations:** 1 Department of Laboratory of Medicine, Ningbo Zhenhai People's Hospital Group, Ningbo, Zhejiang, China; 2 Department of Laboratory of Medicine, Yinzhou District Qiu'ga Central Health Clinic, Ningbo, Zhejiang, China; 3 Ningbo University Health Science Center, Ningbo, Zhejiang, China; 4 Department of Pediatrics, The Affiliated Women and Children's Hospital of Ningbo University, Ningbo, Zhejiang, China; 5 Ningbo Key Laboratory for the Prevention and Treatment of Embryo Original Diseases, The Affiliated Women and Children's Hospital of Ningbo University, Ningbo, Zhejiang, China; 6 Ningbo Key Laboratory of Genomic Medicine and Birth Defects Prevention, The Affiliated Women and Children's Hospital of Ningbo University, Ningbo, Zhejiang, China

**Keywords:** 25-hydroxyvitamin D, community health, COVID-19 pandemic, nutritional epidemiology, preschool children, seasonality, supplementation, vitamin D status

## Abstract

**Background:**

Preschool children are particularly vulnerable to vitamin D (VD) insufficiency owing to rapid skeletal development and limited dietary sources. Although COVID-19 restrictions reduced outdoor activity and theoretically increased deficiency risk, emerging evidence suggests that behavioral adaptations—especially intensified supplementation—may have offset these effects. Yet serial cross-sectional data spanning the pre-, during-, and post-pandemic periods in this population remain scarce, and age-specific vulnerabilities and seasonal disparities are poorly characterized. This study evaluated temporal trends in serum 25-hydroxyvitamin D [25(OH)D] status across three pandemic phases in children under 6 years, examining age-stratified associations and seasonal variations.

**Methods:**

In a community-based cross-sectional study, serum 25(OH)D was quantified by liquid chromatography-mass spectrometry in 6,681 children (<6 years). Participants were stratified into Pre-COVID (2019), During-COVID (2020–2022), and Post-COVID (2022–2023) phases. Multivariable linear and logistic regression models, adjusted for sex, age, and season, assessed pandemic phase associations on continuous 25(OH)D and sufficiency rates (≥30 ng/mL). Subgroup and interaction analyses were conducted by age and season.

**Results:**

Median 25(OH)D rose progressively across phases (30.3 → 34.7 → 37.1 ng/mL; *P* for trend <0.001). Post-COVID versus pre-COVID, fully adjusted models showed a 5.24 ng/mL increase (95% CI 4.51–5.97) and 2.25-fold higher odds of sufficiency (95% CI 1.93–2.63). Infants (<1 year) exhibited the greatest gain (*Δ* + 6.0 ng/mL), whereas children aged 3–6 years showed minimal change (*P* for interaction <0.001). Spring samples demonstrated the largest increase (25.5 → 38.3 ng/mL), with the previously pronounced seasonal trough markedly attenuated.

**Conclusion:**

Despite reduced outdoor activity, VD status improved significantly during and after the pandemic, potentially reflecting increased supplementation practices and altered health behaviors, though these factors were not directly measured. The greatest gains occurred in infants and during spring, suggesting that targeted supplementation may help counteract sunlight deprivation. These findings generate the hypothesis that public health strategies promoting age-specific VD supplementation could help sustain such improvements; however, prospective interventional studies are warranted to confirm these observations and establish causality.

## Introduction

Vitamin D (VD) plays pivotal roles in pediatric bone mineralization, immune regulation, and cellular differentiation (1). Globally, VD insufficiency (serum 25-hydroxyvitamin D [25(OH)D] < 30 ng/mL) affects 30–80% of children, with higher prevalence in winter and among infants due to limited sunlight exposure and dietary sources (2, 3). The COVID-19 pandemic precipitated unprecedented lifestyle disruptions, including prolonged indoor confinement and reduced outdoor activity, which theoretically increased VD deficiency risk (4). Paradoxically, emerging evidence suggests behavioral adaptations—particularly intensified supplementation—may have counterbalanced these effects in specific populations (5, 6).

Recent studies report heterogeneous VD trends during the pandemic. In unsupplemented school-aged cohorts, lockdowns exacerbated seasonal VD declines due to restricted UVB exposure (7, 8). Conversely, supplemented adult populations demonstrated stable or increased 25(OH)D levels, highlighting supplementation’s protective role (6). However, critical knowledge gaps persist regarding preschool children—a developmentally vulnerable group with distinct nutritional dependencies.

Current evidence remains limited by several methodological constraints. Firstly, inadequate age stratification persists as most studies aggregate children beyond 6 years of age (9), obscuring critical infancy-specific vulnerabilities. Additionally, temporal limitations exist wherein few analyses span the pre-, during-, and post-pandemic continuum (10), preventing comprehensive assessment of sustained trends. Furthermore, many investigations overlook essential confounders—particularly seasonal variation and demographic shifts during healthcare disruptions (11)—potentially biasing effect estimates.

Our study addresses these gaps through a large-scale, seasonally adjusted analysis of 6,681 preschool children. We hypothesize that pandemic-induced behavioral changes---potentially including altered VD supplementation practices---may be associated with marked changes in VD trajectories in this population, with effects potentially modulated by age and season.

## Materials and methods

This study followed the STROBE (Strengthening the Reporting of Observational Studies in Epidemiology) guidelines for the transparent reporting of observational research.

### Study design and population

This cross-sectional study utilized de-identified electronic health records from community-based health examinations conducted across Ningbo, Zhejiang Province, China. Serum 25(OH)D testing was not a universal screening mandate but was performed at the discretion of attending physicians or upon parental request during routine check-ups, typically when children presented with risk factors for vitamin D deficiency (e.g., winter season, limited outdoor activity, or clinical suspicion of rickets). Consequently, the study population represents a selected sample of children who underwent 25(OH)D testing rather than a systematically screened cohort. The clinical criteria for ordering 25(OH)D testing were based on standard community health examination protocols that remained unchanged during the study period. The demographic composition of children presenting for examination changed substantially in a pattern consistent with pandemic-related disruptions in healthcare utilization, although we lack formal quantitative data to confirm this interpretation. We analyzed 6,681 preschool children (<6 years) with documented serum 25(OH)D measurements, stratified into three pandemic phases:

Pre-COVID: January 2019–December 2019 (*n* = 2,563).
During-COVID: January 2020–November 2022 (*n* = 2,814).
Post-COVID: December 2022–December 2023 (*n* = 1,304).

Children with chronic conditions affecting vitamin D metabolism (renal/liver disease, malabsorption syndromes) or using bone-altering medications (glucocorticoids, anticonvulsants) were excluded.

### Data collection and laboratory analysis

Blood samples collected during morning community visits (08:00–10:00 a.m.) were analyzed using liquid chromatography-mass spectrometry (LC–MS; AB SCIEX Triple Quad 4500MD) with protein precipitation and hexane extraction. Quality assurance included daily calibration with NIST-traceable standards (inter-assay coefficient of variation <5%). Vitamin D status was classified as: severe deficiency (<10 ng/mL), deficiency (10–19.9 ng/mL), insufficiency (20–29.9 ng/mL), or sufficiency (≥30 ng/mL), based on the 2022 Chinese Guideline for Clinical Practice of Vitamin D Nutrition in Children and the widely adopted pediatric classification standards used in mainland Chinese studies.

Demographic covariates (age groups: <1y, 1–3y, 3–6y; sex), and sampling season (spring: March–May; summer: June–August; autumn: September–November; winter: December–February) were recorded. The dataset comprised children who underwent serum 25(OH)D testing during routine community health examinations. Testing was not universally mandated but was performed based on clinical indication or parental request. The proportion of children tested varied by age and pandemic phase, reflecting differential healthcare utilization patterns. To mitigate confounding from this demographic shift, all regression models adjusted for age group (<1y, 1–3y, 3–6y) as a categorical covariate, and age-stratified subgroup analyses were conducted.

### Statistical analysis

Analyses were performed in R (v4.3.0; R Foundation) and Free Statistics (v2.4.0; Beijing Free Clinical Medical Technology). Continuous variables with non-normal distribution were summarized as median (interquartile range); categorical variables as frequencies (percentages). Group comparisons employed Kruskal-Wallis tests for continuous variables and χ^2^ tests for categorical variables.

Multivariable linear regression models evaluated the independent association between pandemic phases and continuous 25(OH)D levels. Binary logistic regression assessed the odds of vitamin D sufficiency (≥30 ng/mL). Both models were adjusted for sex, age group, and season, with sequential adjustment: Model 1 (unadjusted), Model 2 (adjusted for sex and age), and Model 3 (fully adjusted for sex, age, and season). Trend analyses assigned ordinal values to pandemic phases to evaluate linear trends.

Subgroup analyses stratified by sex, age group, and season were conducted, with interaction terms testing for effect modification. Sensitivity analysis excluded data from December 2022 to June 2023 to address potential confounding during the immediate post-lockdown transition. Statistical significance was defined as two-tailed *p* < 0.05, with missing data (<2% across variables) handled via complete-case analysis.

## Results

### Baseline characteristics

Baseline characteristics of the 6,681 included children are presented in Table 1. There were 2,563 children in the pre-COVID period, 2,814 in the during-COVID period, and 1,304 in the post-COVID period. Sex distribution was comparable across the three periods (*p* = 0.316). Significant differences were observed in age structure (*p* < 0.001): the proportion of infants aged <1 year increased markedly from 46.6% in the pre-COVID period to 70.6% during COVID and 83.0% post-COVID, while the proportion of children aged 3–6 years decreased correspondingly. The distribution of sampling season also differed significantly among groups (*p* < 0.001), with a higher proportion of cases occurring in winter in the post-COVID period.

**Table 1:** Baseline characteristics of the study population stratified by COVID-19 pandemic periods.

Variables	Total (*n* = 6,681)	Pre-COVID (*n* = 2,563)	During-COVID (*n* = 2,814)	Post-COVID (*n* = 1,304)	*P*
Sex, *n* (%)					0.316
Male	3,463 (51.8)	1,358 (53.0)	1,443 (51.3)	662 (50.8)	
Female	3,218 (48.2)	1,205 (47.0)	1,371 (48.7)	642 (49.2)	
Age, *n* (%)					<0.001
<1 Year	4,262 (63.8)	1,194 (46.6)	1986 (70.6)	1,082 (83.0)^a^	
1–3 Years	1,638 (24.5)	983 (38.4)	463 (16.5)	192 (14.7)	
3–6 Years	781 (11.7)	386 (15.1)	365 (13.0)	30 (2.3)	
Season, *n* (%)					<0.001
Spring	1934 (28.9)	709 (27.7)	810 (28.8)	415 (31.8)	
Summer	2,104 (31.5)	841 (32.8)	894 (31.8)	369 (28.3)	
Autumn	1,513 (22.6)	642 (25.0)	656 (23.3)	215 (16.5)	
Winter	1,130 (16.9)	371 (14.5)	454 (16.1)	305 (23.4)	

a The marked increase in infant proportion post-COVID likely reflects pandemic-related changes in healthcare utilization, consistent with institutional records indicating that infant visits (tied to immunization schedules) were maintained while older preschool visits declined.

### Vitamin D levels and nutritional status

**Figure 1:**
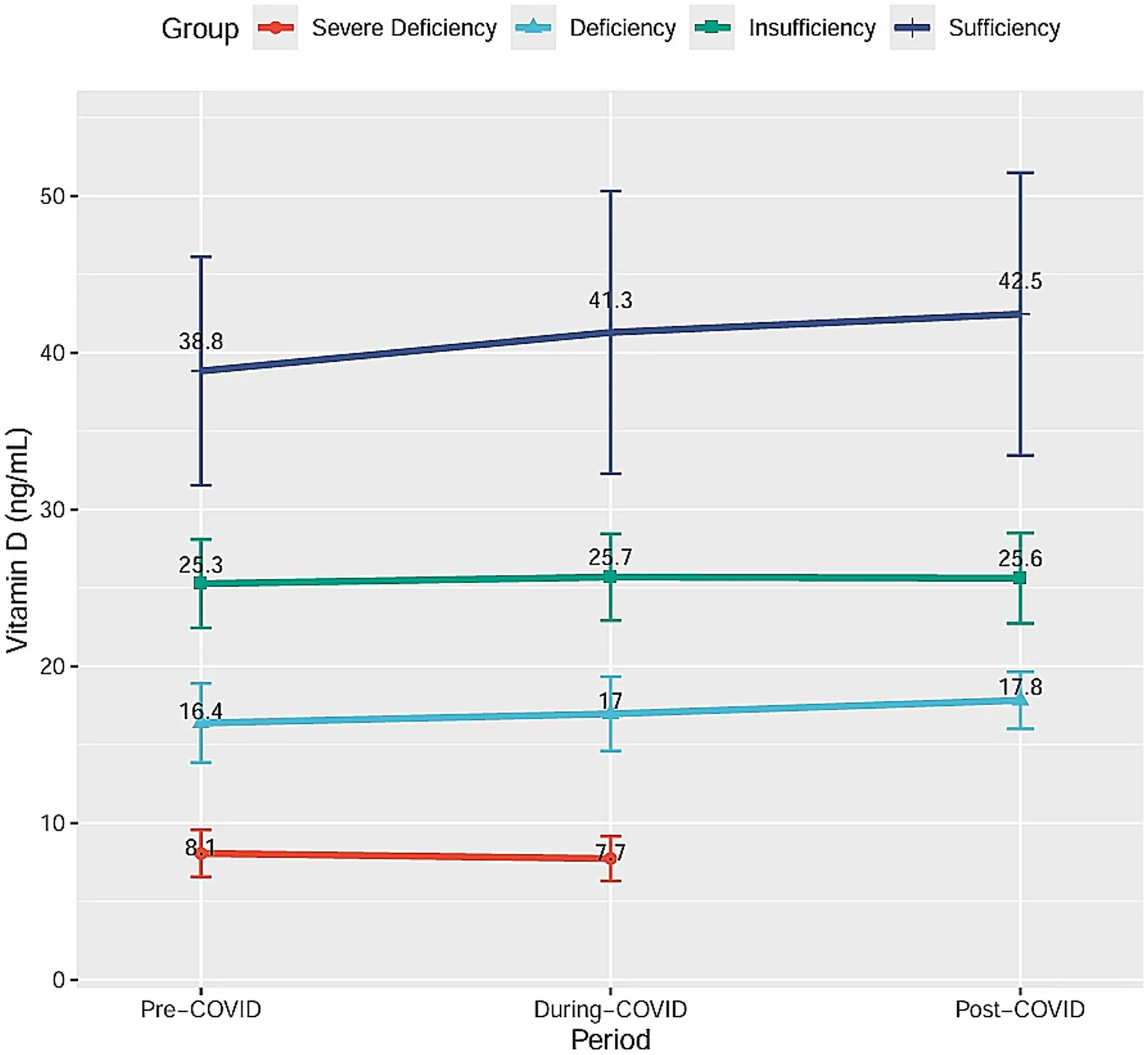
Temporal trends of serum vitamin D concentrations according to vitamin D nutritional status across pre-COVID, during-COVID, and post-COVID periods.

**Figure 2:**
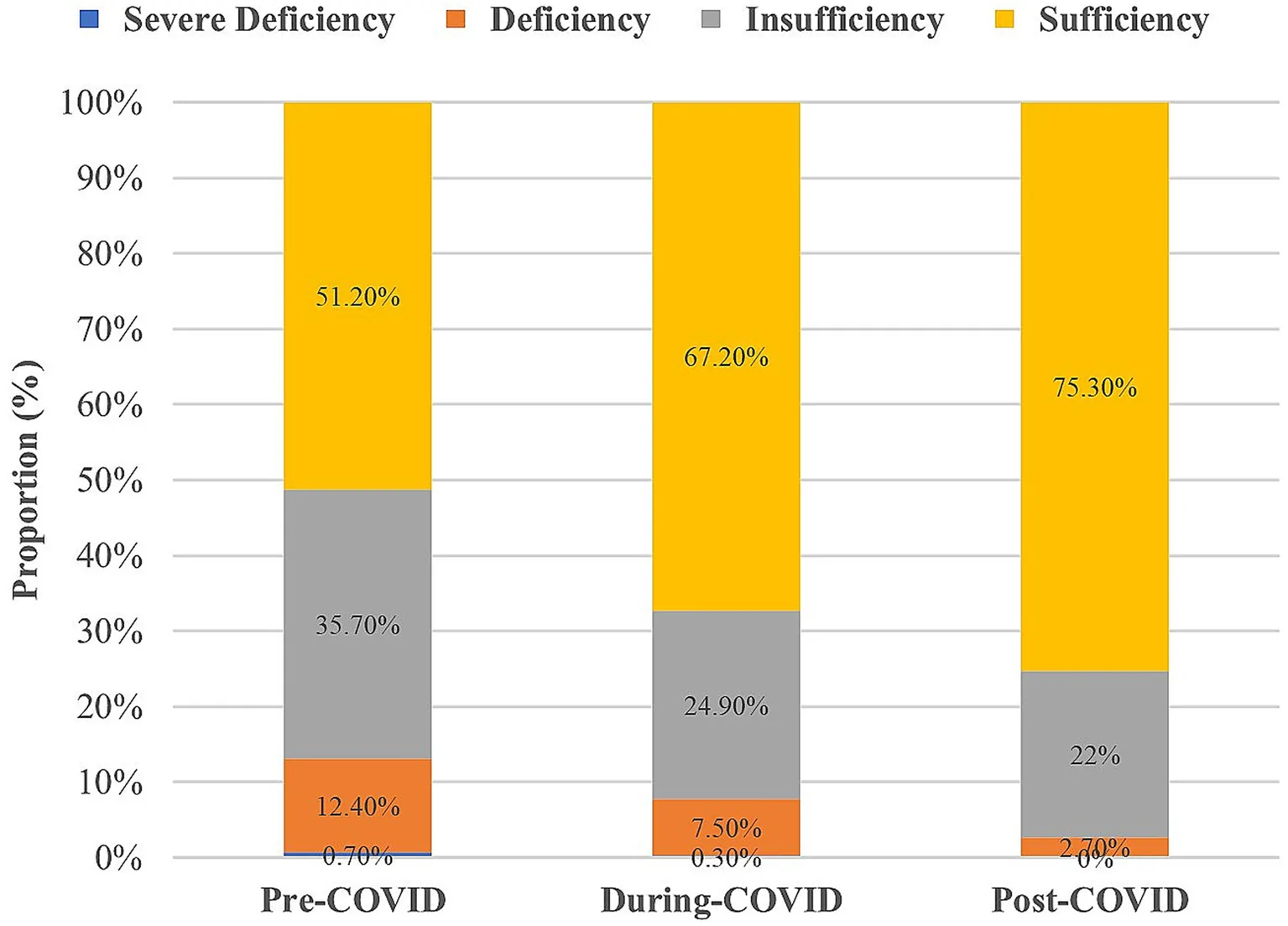
Proportional distribution of vitamin D nutritional status across pre-COVID, during-COVID, and post-COVID periods.

As shown in Table 2 and Figures 1, 2, serum vitamin D status improved progressively across the three periods. Median serum 25(OH)D concentration increased from 30.3 ng/mL (IQR: 23.7–37.2) in the pre-COVID period to 34.7 ng/mL (IQR: 27.4–42.5) during COVID and 37.1 ng/mL (IQR: 30.0–44.8) post-COVID (*P* for trend <0.001).

**Table 2:** Vitamin D levels and nutritional status stratified by COVID-19 pandemic periods.

Variables	Total (*n* = 6,681)	Pre-COVID (*n* = 2,563)	During-COVID (*n* = 2,814)	Post-COVID (*n* = 1,304)	*P*
Vitamin D, Median (IQR), ng/mL	33.4 (26.2, 41.2)	30.3 (23.7, 37.2)	34.7 (27.4, 42.5)	37.1 (30.0, 44.8)	<0.001
Vitamin D status, *n* (%)					<0.001
Severe deficiency	26 (0.4)	18 (0.7)	8 (0.3)	0 (0)	
Deficiency	565 (8.5)	318 (12.4)	212 (7.5)	35 (2.7)	
Insufficiency	1903 (28.5)	914 (35.7)	702 (24.9)	287 (22.0)	
Sufficiency	4,187 (62.7)	1,313 (51.2)	1892 (67.2)	982 (75.3)	

According to nutritional status classification, the proportion of children with vitamin D sufficiency (≥30 ng/mL) rose from 51.2% pre-COVID to 67.2% during COVID and 75.3% post-COVID. Correspondingly, the prevalence of vitamin D deficiency declined from 12.4 to 2.7%, and severe deficiency decreased from 0.7 to 0% by the post-COVID period. These trends were visualized clearly in the temporal trend graph and proportional distribution chart (Figures 1, 2).

### Independent association of pandemic periods on vitamin D status

Multivariable linear regression models (Table 3) were used to evaluate the independent association of pandemic periods on continuous vitamin D levels after adjusting for sex, age, and season. Compared with the pre-COVID period, vitamin D concentrations were significantly higher in the during-COVID (adjusted *β* = 3.35, 95% CI: 2.77–3.93, *p* < 0.001) and post-COVID (adjusted β = 5.24, 95% CI: 4.51–5.97, *p* < 0.001) periods. A significant linear increasing trend was detected across periods (adjusted β = 2.72, 95% CI: 2.36–3.08, *p* < 0.001).

**Table 3:** Multivariable linear regression models for the association of pandemic periods on vitamin D levels (continuous).

Variable	Model 1 *β* coefficient (95%CI)	*P*	Model 2 *β* coefficient (95%CI)	*P*	Model 3 *β* coefficient (95%CI)	*P*
Pre-COVID	0 (Ref)		0 (Ref)		0 (Ref)	
During-COVID	4.48 (3.89 ~ 5.06)	<0.001	3.29 (2.71 ~ 3.87)	<0.001	3.35 (2.77 ~ 3.93)	<0.001
Post-COVID	7.10 (6.37 ~ 7.83)	<0.001	4.97 (4.24 ~ 5.71)	<0.001	5.24 (4.51 ~ 5.97)	<0.001
Linear trend	3.68 (3.33 ~ 4.04)	<0.001	2.59 (2.23 ~ 2.95)	<0.001	2.72 (2.36 ~ 3.08)	<0.001

Model 1: crude model, unadjusted. Model 2: adjusted for sex and age. Model 3: fully adjusted model, adjusted for sex, age, and season.

Logistic regression analyses (Table 4) further confirmed these findings using vitamin D sufficiency (≥30 ng/mL vs. <30 ng/mL) as the binary outcome. In the fully adjusted model, children in the during-COVID period had 1.70-fold higher odds of vitamin D sufficiency (OR = 1.70, 95% CI: 1.51–1.92, *p* < 0.001), and those in the post-COVID period had 2.25-fold higher odds (OR = 2.25, 95% CI: 1.93–2.63, *p* < 0.001) compared with the pre-COVID group. A significant linear trend for improved sufficiency rates was also observed (adjusted OR = 1.54, 95% CI: 1.43–1.66, *p* < 0.001).

**Table 4:** Logistic regression analysis of the association between COVID-19 pandemic periods and vitamin D sufficiency status (<30 ng/mL vs. ≥30 ng/mL).

Variable	Model 1 OR (95%CI)	*P*	Model 2 OR (95%CI)	*P*	Model 3 OR (95%CI)	*P*
Pre-COVID	1 (Ref)		1 (Ref)		1 (Ref)	
During-COVID	1.95 (1.75 ~ 2.18)	<0.001	2 (1.79 ~ 2.24)	<0.001	1.7 (1.51 ~ 1.92)	<0.001
Post-COVID	2.9 (2.5 ~ 3.37)	<0.001	3.1 (2.67 ~ 3.61)	<0.001	2.25 (1.93 ~ 2.63)	<0.001
Linear trend	1.76 (1.64 ~ 1.89)	<0.001	1.81 (1.69 ~ 1.95)	<0.001	1.54 (1.43 ~ 1.66)	<0.001

Model 1: crude model, unadjusted. Model 2: adjusted for sex and age. Model 3: fully adjusted model, adjusted for sex, age, and season.

### Subgroup and interaction analyses

**Figure 3:**
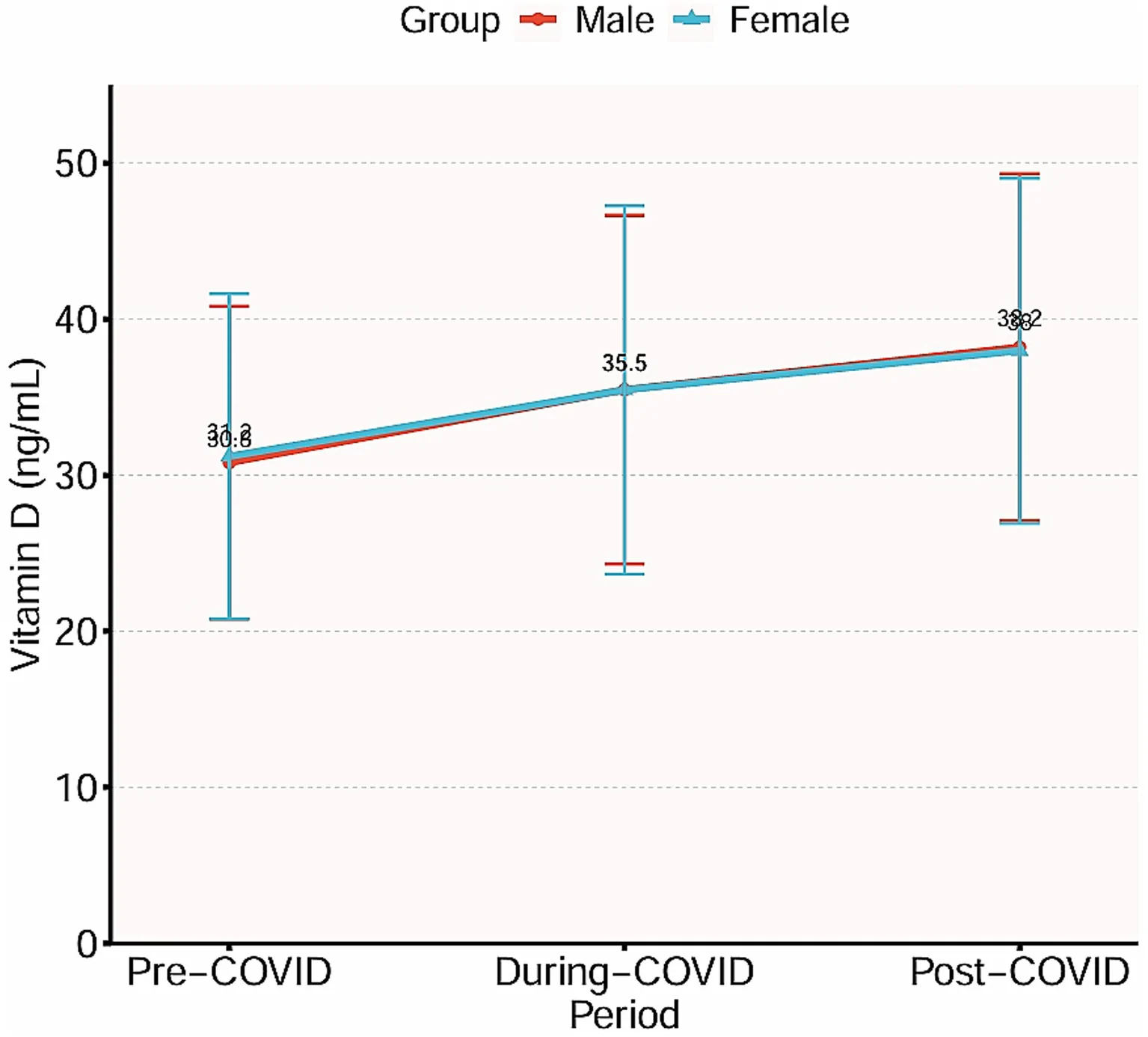
Trends in serum vitamin D levels across pandemic periods stratified by sex.

**Figure 4:**
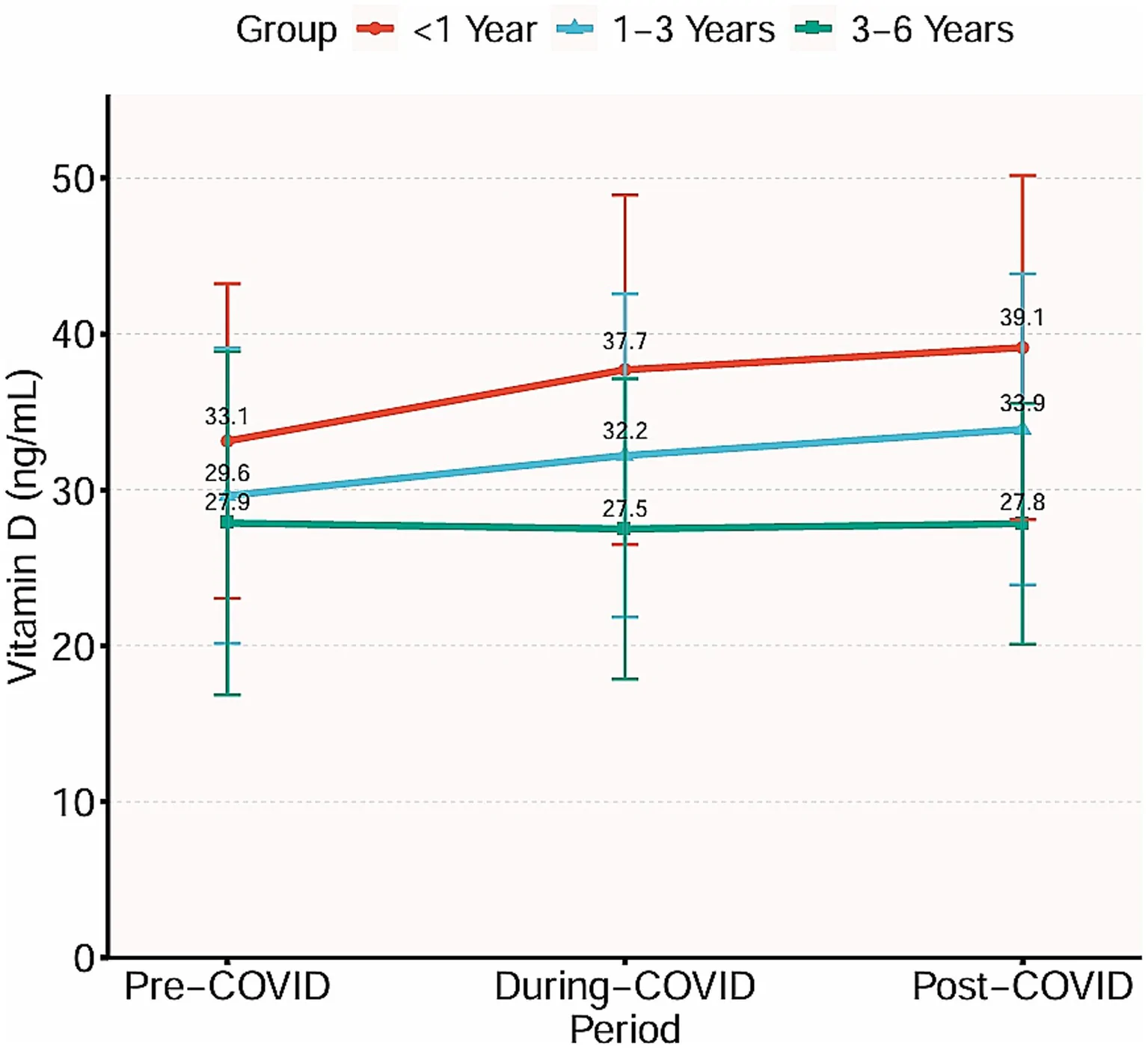
Trends in serum vitamin D levels across pandemic periods stratified by age group.

**Figure 5:**
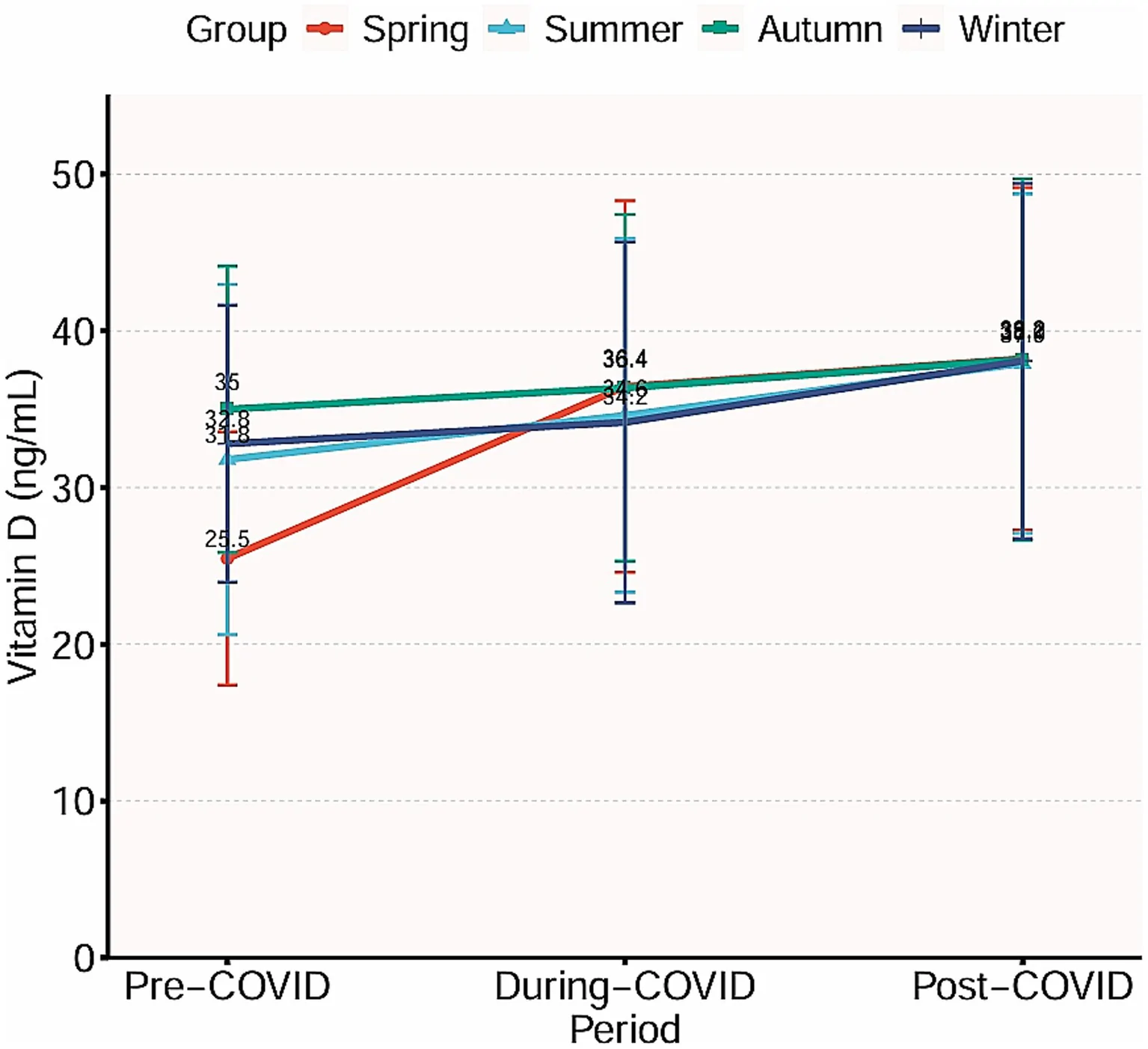
Trends in serum vitamin D levels across pandemic periods stratified by season.

Subgroup analyses stratified by sex, age group, and season were conducted to identify potential effect modifications (Figures 3–5).

For sex, increases in vitamin D levels and sufficiency rates across periods were similar in boys and girls, with no significant interaction (*P* for interaction = 0.34; Figure 3).

By age group, the most pronounced improvement was observed in infants <1 year old, whose vitamin D levels rose consistently and whose odds of sufficiency increased most markedly (all *p* < 0.001). In contrast, children aged 3–6 years showed minimal changes across periods. A significant interaction between age group and pandemic period was detected (*p* < 0.001; Figure 4).

Regarding season, children tested in spring exhibited the most dramatic elevation in vitamin D levels and sufficiency rates, with the lowest baseline levels in the pre-COVID period increasing to levels comparable to other seasons post-COVID. Children tested in autumn or winter showed no significant improvements. A significant interaction was identified between season and pandemic period (*p* < 0.001; Figure 5).

**Figure 6:**
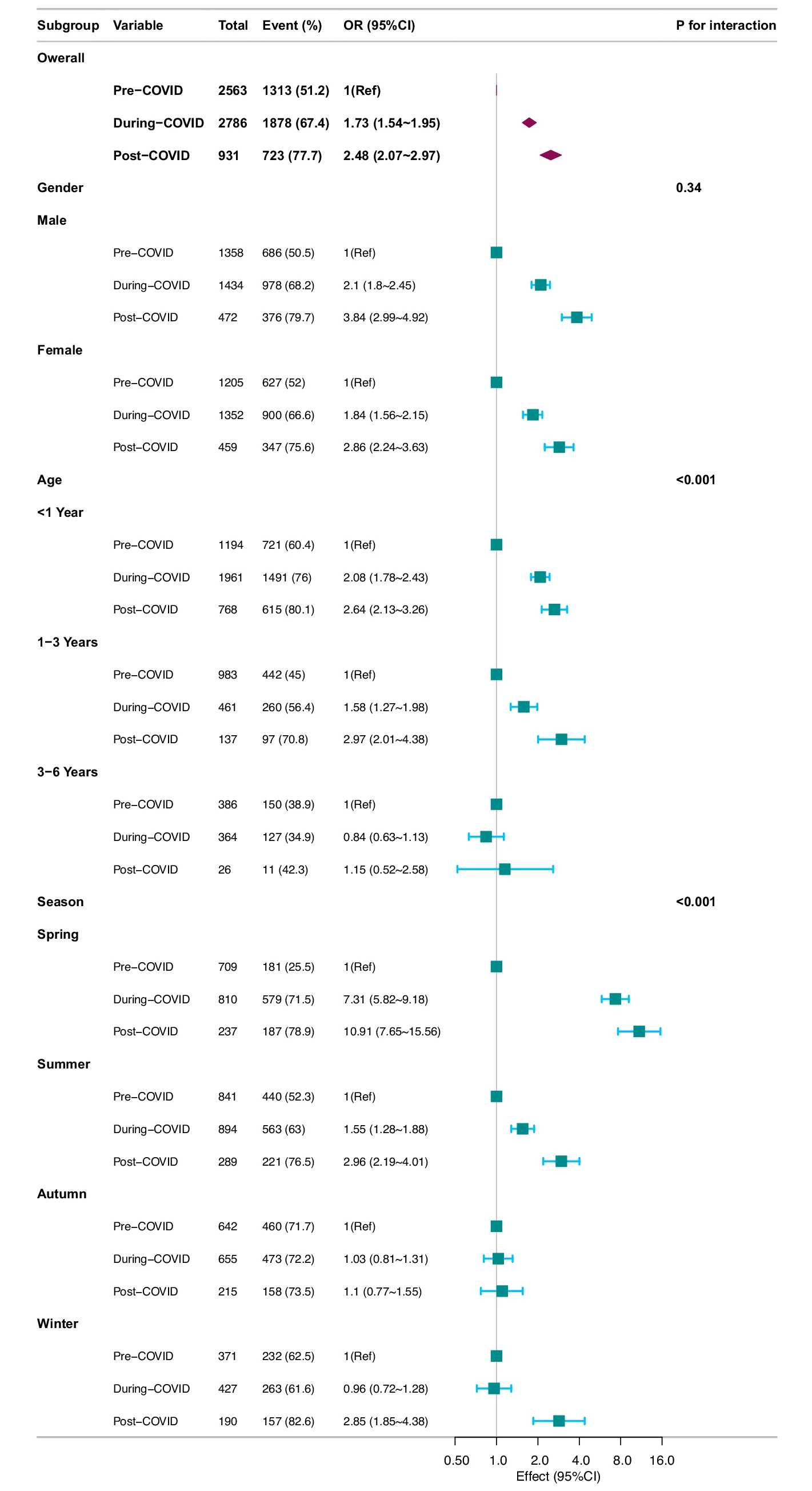
Subgroup analyses for the association between COVID-19 pandemic periods and vitamin D sufficiency status, stratified by sex, age group, and season.

Forest plot analyses (Figure 6) demonstrated strong positive associations among infants and children tested in spring, whereas no significant improvements were observed in older children or those tested in autumn or winter.

### Sensitivity analysis

A sensitivity analysis excluding data from the immediate post-lockdown transition period (December 2022 – June 2023) to minimize potential confounding from the initial infection wave yielded results consistent with the primary analysis using the full dataset (see Supplementary Tables S1–S4; Supplementary Figure S1). Additionally, age-stratified multivariable linear and logistic regression analyses were performed to address the marked demographic shift across pandemic periods; detailed estimates are provided in Supplementary Tables S5, S6, respectively.

## Discussion

This study provides evidence that the COVID-19 pandemic phases were significantly associated with progressive improvements in vitamin D (VD) status among preschool children, even after rigorous adjustment for key confounders. Our findings are consistent with a complex interplay between pandemic-related societal shifts, age-specific vulnerabilities, and the mitigation of pre-existing seasonal VD disparities.

The most striking observation was the stepwise increase in serum 25-hydroxyvitamin D concentrations and sufficiency rates (≥30 ng/mL) across pre-COVID, during-COVID, and post-COVID periods. This trend persisted after controlling for age, sex, and season—factors exhibiting significant baseline imbalances. The magnitude of improvement (adjusted *β* = 5.24 ng/mL for post-COVID vs. pre-COVID; OR = 2.25 for sufficiency) is clinically meaningful, and may potentially reflect multifaceted behavioral changes, although we did not directly measure supplementation, dietary intake, or outdoor activity. We speculate that increased parental awareness of immune health benefits during the pandemic may have contributed to higher VD supplementation rates; however, this remains hypothetical in the absence of direct behavioral data. This speculation aligns with global reports of escalated supplement use (12–15). Paradoxically, although lockdowns were associated with reduced outdoor activity at the population level, our cohort showed substantial VD gains, which may suggest that supplementation may have outweighed sunlight deprivation, though residual confounding from unmeasured behavioral factors cannot be excluded. This finding is consistent with reports from adult populations where widespread and intensified supplementation was reported to be associated with increased mean VD levels during the pandemic, as observed in Irish hospital samples and US single-center studies (5, 6). Conversely, in populations without systematic supplementation or with low supplementation rates, such as many school-aged children and adolescents or general populations in certain countries, lockdowns and reduced outdoor activity typically resulted in decreased VD levels or exacerbated seasonal winter/spring declines (7, 16). This contrast is consistent with the hypothesis that a critical determinant of VD status during periods of restricted sunlight exposure may be the adoption and adequacy of supplementation practices, rather than solely the deprivation itself.

Notably, the pandemic appeared to modify traditional VD seasonality patterns. Pre-COVID spring samples showed the lowest VD levels (25.5 ng/mL), consistent with winter sunlight deficits (17). Post-COVID, the previously pronounced seasonal trough in 25(OH)D levels was markedly attenuated, with spring levels becoming comparable to those observed in other seasons. This observed trend, characterized by an overall upward shift in annual 25(OH)D levels and a moderated decline during winter, aligns with patterns reported in certain adult populations (5, 18). It is plausible that this phenomenon is linked to increased supplement usage and heightened health awareness during the pandemic, suggesting a potential positive public health impact (6, 19). However, prospective studies are warranted to confirm these observations and elucidate the underlying mechanisms.

Age-stratified analyses revealed critical heterogeneity. Infants (<1 year) exhibited the most dramatic gains (*Δ* + 6.0 ng/mL post-COVID vs. pre-COVID), potentially reflecting targeted supplementation in this vulnerable group, though unmeasured confounders cannot be excluded. Conversely, older preschoolers (3–6 years) showed negligible improvement, possibly due to lower supplementation adherence and persistent indoor behaviors (20, 21). These observations suggest that age-tailored interventions may warrant consideration in future research. Notably, this cohort demonstrated no sex-based differences in 25(OH)D concentrations, aligning with observations in other preschool populations (22). This finding suggests broad equilibrium in vitamin D nutritional status between males and females within this developmental stage and environmental context.

Our findings are biologically plausible in light of accumulating mechanistic and clinical data on vitamin D and respiratory infections. Vitamin D modulates both innate and adaptive immunity by enhancing Toll-like receptor–driven antimicrobial responses, up-regulating cathelicidin and *β*-defensins, promoting autophagy in macrophages, and dampening pro-inflammatory Th1/Th17 cytokine production while supporting regulatory T-cell and antibody responses (23, 24). Through these pathways, adequate 25(OH)D status may both improve viral clearance and limit the hyperinflammatory responses that characterize severe COVID-19 and MIS-C (25).

Pediatric studies further suggest that low vitamin D levels are common among children with COVID-19 and are associated with more pronounced inflammatory responses and greater clinical severity, including higher rates of fever and elevated CRP, fibrinogen, D-dimer, and lymphopenia (26). A recent randomized controlled trial in hospitalized children with moderate COVID-19 reported that daily vitamin D supplementation tended to reduce progression to more intensive respiratory support and mortality, with no safety concerns, although sample size was limited (27). Narrative and systematic reviews therefore converge on the view that maintaining vitamin D sufficiency is a reasonable, low-risk strategy to support host defense against SARS-CoV-2 in children, while definitive effect sizes remain uncertain (28).

Within this context, the marked improvement in vitamin D status—particularly among infants—in our cohort may represent a modifiable resilience factor during and beyond the pandemic. However, as an observational study without infection outcomes, our data cannot establish that higher 25(OH)D directly reduced SARS-CoV-2 susceptibility or disease severity. Future prospective and interventional studies linking longitudinal vitamin D trajectories to virologic and clinical endpoints in preschool populations are needed to clarify whether the gains documented here translate into measurable reductions in respiratory morbidity, including COVID-19 and other viral RTIs.

### Limitations and strengths

The single-center design may limit generalizability, though our large sample (*n* = 6,681) enhances robustness. Residual confounding from unmeasured factors (diet, supplement dosage, precise sunlight exposure) cannot be excluded. However, rigorous adjustment for key covariates, sensitivity analyses, and consistent linear/logistic models enhance the reliability of observed associations. The dramatic shift in age structure toward infants post-COVID, while analytically controlled via multivariable adjustment and age-stratified subgroup analyses, remains a significant limitation. This compositional change likely reflects altered healthcare-seeking behaviors during the pandemic, although we lack formal quantitative data to confirm age-specific healthcare utilization patterns, and may partially explain the attenuation of seasonal disparities, given that infants typically receive more standardized year-round supplementation. Consequently, the generalizability of our seasonal findings to older preschool populations is uncertain, and the observed attenuation of seasonal differences may be partly attributable to this demographic shift rather than solely to pandemic-related behavioral changes. The post-COVID 3–6-year subgroup comprised only 30 children (2.3%), rendering estimates in this stratum statistically unstable. Future studies should employ fixed-cohort designs or pre-specified age-stratified sampling to ensure demographic stability across periods.

## Conclusion

The COVID-19 pandemic was associated with significant improvements in preschool children’s VD status, most profoundly in infants and during spring. These changes may potentially reflect heightened supplementation practices and altered health behaviors, although these factors were not directly measured. Notably, these changes were accompanied by a marked attenuation of the pre-pandemic seasonal trough in 25(OH)D levels. These findings raise the hypothesis that public health strategies promoting year-round VD supplementation, particularly for older preschoolers, could help sustain such improvements, pending confirmation by prospective interventional studies. Our study illuminates the potential for positive nutritional legacies emerging from global health crises.

## Data Availability

The original contributions presented in the study are included in the article/Supplementary material, further inquiries can be directed to the corresponding authors.
