# Supplementary material for: Pandemic-related changes in seasonal vitamin D disparities among preschool children: a community-based cross-sectional study
Source: Front Nutr. 2026 Aug 17;13:1893922. doi: 10.3389/fnut.2026.1893922 (PMC13526663; doi:10.3389/fnut.2026.1893922)
Supplement: Supplementary file 1 [file Data_Sheet_1.zip › Supplementary Table 6.docx]

**S6.** Age-stratified logistic regression analysis of the association between COVID-19 pandemic periods and vitamin D sufficiency status (<30 ng/mL vs. ≥30 ng/mL)

| Age group | Variable | Model 1  OR (95%CI) | *P* | Model 2  OR (95%CI) | *P* | Model 3  OR (95%CI) | *P* |
| --- | --- | --- | --- | --- | --- | --- | --- |
| <1 Year | Pre-COVID | 1(Ref) |  | 1(Ref) |  | 1(Ref) |  |
|  | During-COVID | 2.05 (1.76~2.4) | <0.001 | 2.05 (1.76~2.39) | <0.001 | 2.09 (1.79~2.45) | <0.001 |
|  | Post-COVID | 2.38 (1.97~2.86) | <0.001 | 2.38 (1.97~2.86) | <0.001 | 2.47 (2.05~2.98) | <0.001 |
|  | Linear trend | 1.59 (1.45~1.75) | <0.001 | 1.59 (1.45~1.75) | <0.001 | 1.62 (1.47~1.78) | <0.001 |
| 1-3 Years | Pre-COVID | 1(Ref) |  | 1(Ref) |  | 1(Ref) |  |
|  | During-COVID | 1.57 (1.25~1.96) | <0.001 | 1.57 (1.26~1.96) | <0.001 | 1.52 (1.21~1.91) | <0.001 |
|  | Post-COVID | 2.18 (1.58~3.01) | <0.001 | 2.19 (1.59~3.02) | <0.001 | 2.6 (1.87~3.63) | <0.001 |
|  | Linear trend | 1.5 (1.3~1.74) | <0.001 | 1.51 (1.31~1.74) | <0.001 | 1.58 (1.37~1.83) | <0.001 |
| 3-6 Years | Pre-COVID | 1(Ref) |  | 1(Ref) |  | 1(Ref) |  |
|  | During-COVID | 0.84 (0.62~1.13) | 0.249 | 0.84 (0.63~1.13) | 0.258 | 0.93 (0.68~1.26) | 0.623 |
|  | Post-COVID | 0.91 (0.42~1.97) | 0.812 | 0.9 (0.42~1.95) | 0.788 | 1 (0.46~2.18) | 0.995 |
|  | Linear trend | 0.88 (0.68~1.13) | 0.315 | 0.88 (0.68~1.13) | 0.316 | 0.95 (0.73~1.24) | 0.708 |

Model 1: crude model, unadjusted. Model 2: adjusted for sex and age. Model 3: fully adjusted model, adjusted for sex, age, and season.
