# Supplementary material for: Pandemic-related changes in seasonal vitamin D disparities among preschool children: a community-based cross-sectional study
Source: Front Nutr. 2026 Aug 17;13:1893922. doi: 10.3389/fnut.2026.1893922 (PMC13526663; doi:10.3389/fnut.2026.1893922)
Supplement: Supplementary file 1 [file Data_Sheet_1.zip › Supplementary Table 5.docx]

**S5.** Age-stratified multivariable linear regression models for the effect of pandemic periods on continuous vitamin D levels

| Age group | Variable | Model 1  β confidence ( 95%CI) | *P* | Model 2  adjusted β (95% CI) | *P* | Model 3  adjusted β (95% CI) | *P* |
| --- | --- | --- | --- | --- | --- | --- | --- |
| <1 Year | Pre-COVID | 0(Ref) |  | 0(Ref) |  | 0(Ref) |  |
|  | During-COVID | 4.57 (3.79~5.35) | <0.001 | 4.57 (3.79~5.35) | <0.001 | 4.62 (3.85~5.4) | <0.001 |
|  | Post-COVID | 5.99 (5.1~6.89) | <0.001 | 5.99 (5.1~6.88) | <0.001 | 6.15 (5.26~7.05) | <0.001 |
|  | Linear trend | 3.03 (2.59~3.48) | <0.001 | 3.03 (2.58~3.48) | <0.001 | 3.12 (2.67~3.56) | <0.001 |
| 1-3 Years | Pre-COVID | 0(Ref) |  | 0(Ref) |  | 0(Ref) |  |
|  | During-COVID | 2.58 (1.49~3.66) | <0.001 | 2.58 (1.49~3.66) | <0.001 | 2.32 (1.27~3.38) | <0.001 |
|  | Post-COVID | 4.25 (2.74~5.77) | <0.001 | 4.26 (2.74~5.77) | <0.001 | 4.85 (3.36~6.34) | <0.001 |
|  | Linear trend | 2.25 (1.57~2.93) | <0.001 | 2.26 (1.57~2.94) | <0.001 | 2.4 (1.73~3.06) | <0.001 |
| 3-6 Years | Pre-COVID | 0(Ref) |  | 0(Ref) |  | 0(Ref) |  |
|  | During-COVID | -0.38 (-1.85~1.1) | 0.617 | -0.36 (-1.84~1.11) | 0.629 | -0.09 (-1.56~1.38) | 0.905 |
|  | Post-COVID | -0.04 (-3.86~3.78) | 0.984 | -0.08 (-3.91~3.74) | 0.966 | -0.06 (-3.87~3.74) | 0.974 |
|  | Linear trend | -0.25 (-1.52~1.01) | 0.695 | -0.25 (-1.52~1.01) | 0.696 | -0.07 (-1.34~1.2) | 0.914 |

Model 1: crude model, unadjusted. Model 2: adjusted for sex and age. Model 3: fully adjusted model, adjusted for sex, age, and season.
