# Supplementary material for: Pandemic-related changes in seasonal vitamin D disparities among preschool children: a community-based cross-sectional study
Source: Front Nutr. 2026 Aug 17;13:1893922. doi: 10.3389/fnut.2026.1893922 (PMC13526663; doi:10.3389/fnut.2026.1893922)
Supplement: Supplementary file 1 [file Data_Sheet_1.zip › Supplementary Tables 1 to 4.docx]

**Table S1.**  Baseline Characteristics Stratified by Pandemic Periods (Sensitivity Analysis Excluding Post-Lockdown Transition: December 2022–June 2023)

| **Variables** | **Total**  **(n = 6280)** | **Pre-COVID**  **(n = 2563)** | **During-COVID**  **(n = 2786)** | **Post-COVID**  **(n = 931)** | ***P*** |
| --- | --- | --- | --- | --- | --- |
| Sex, n (%) |  |  |  |  | 0.379 |
| Male | 3264 (52.0) | 1358 (53) | 1434 (51.5) | 472 (50.7) |  |
| Female | 3016 (48.0) | 1205 (47) | 1352 (48.5) | 459 (49.3) |  |
| Age, n (%) |  |  |  |  | < 0.001 |
| <1 Year | 3923 (62.5) | 1194 (46.6) | 1961 (70.4) | 768 (82.5) |  |
| 1-3 Years | 1581 (25.2) | 983 (38.4) | 461 (16.5) | 137 (14.7) |  |
| 3-6 Years | 776 (12.4) | 386 (15.1) | 364 (13.1) | 26 (2.8) |  |
| Season, n (%) | |  |  |  | 0.001 |
| Spring | 1756 (28.0) | 709 (27.7) | 810 (29.1) | 237 (25.5) |  |
| Summer | 2024 (32.2) | 841 (32.8) | 894 (32.1) | 289 (31) |  |
| Autumn | 1512 (24.1) | 642 (25) | 655 (23.5) | 215 (23.1) |  |
| Winter | 988 (15.7) | 371 (14.5) | 427 (15.3) | 190 (20.4) |  |

**Table S2.** Vitamin D Levels and Nutritional Status Across Pandemic Periods (Sensitivity Analysis Excluding Transition Period).

| **Variables** | **Total**  **(n = 6280)** | **Pre-COVID**  **(n = 2563)** | **During-COVID**  **(n = 2786)** | **Post-COVID**  **(n = 931)** | ***P*** |
| --- | --- | --- | --- | --- | --- |
| Vitamin D, Median (IQR), ng/mL | 33.3 (26.2, 41.0) | 30.3 (23.7, 37.2) | 34.7 (27.4, 42.5) | 37.8 (30.8, 45.2) | < 0.001 |
| Vitamin D Status, n (%) |  |  |  |  | < 0.001 |
| Severe Deficiency | 26 ( 0.4) | 18 (0.7) | 8 (0.3) | 0 (0) |  |
| Deficiency | 548 ( 8.7) | 318 (12.4) | 209 (7.5) | 21 (2.3) |  |
| Insufficiency | 1792 (28.5) | 914 (35.7) | 691 (24.8) | 187 (20.1) |  |
| Sufficiency | 3914 (62.3) | 1313 (51.2) | 1878 (67.4) | 723 (77.7) |  |

**Table S3.** Linear Mixed Models for Pandemic Phase Effects on Continuous Vitamin D Levels (Sensitivity Analysis)

| Variable | Model 1  (β confidence, 95%CI) | *P* | Model 2  (β confidence, 95%CI) | *P* | Model 3  (β confidence, 95%CI) | *P* |
| --- | --- | --- | --- | --- | --- | --- |
| Pre-COVID | 0(Ref) |  | 0(Ref) |  | 0(Ref) |  |
| During-COVID | 4.49 (3.91~5.08) | <0.001 | 3.34 (2.76~3.93) | <0.001 | 3.41 (2.83~3.99) | <0.001 |
| Post-COVID | 7.69 (6.87~8.51) | <0.001 | 5.64 (4.83~6.46) | <0.001 | 5.64 (4.83~6.45) | <0.001 |
| Trend.test | 4 (3.61~4.38) | <0.001 | 2.94 (2.55~3.33) | <0.001 | 2.95 (2.57~3.34) | <0.001 |

Model 1: crude model, unadjusted. Model 2: adjusted for sex and age. Model 3: fully adjusted model, adjusted for sex, age, and season.

**Table S4.** Logistic Regression for Vitamin D Sufficiency Status (Sensitivity Analysis: Post-Lockdown Transition Excluded)

| Variable | Model 1 (OR, 95%CI) | *P* | Model 2 (OR, 95%CI) | *P* | Model 3 (OR, 95%CI) | *P* |
| --- | --- | --- | --- | --- | --- | --- |
| Pre-COVID | 1(Ref) |  | 1(Ref) |  | 1(Ref) |  |
| During-COVID | 1.97 (1.76~2.2) | <0.001 | 1.69 (1.5~1.9) | <0.001 | 1.73 (1.54~1.95) | <0.001 |
| Post-COVID | 3.31 (2.78~3.93) | <0.001 | 2.43 (2.03~2.9) | <0.001 | 2.48 (2.07~2.97) | <0.001 |
| Trend.test | 1.87 (1.73~2.02) | <0.001 | 1.6 (1.47~1.73) | <0.001 | 1.62 (1.49~1.76) | <0.001 |

Model 1: crude model, unadjusted. Model 2: adjusted for sex and age. Model 3: fully adjusted model, adjusted for sex, age, and season.
